# Functional Characteristics Analysis of Dehydrins in *Larix kaempferi* under Osmotic Stress

**DOI:** 10.3390/ijms22041715

**Published:** 2021-02-09

**Authors:** Xuechun Wang, Meng Zhang, Baohui Xie, Xiangning Jiang, Ying Gai

**Affiliations:** 1 College of Biological Sciences and Biotechnology, Beijing Forestry University, Beijing 100083, China; wangxuechun0717@126.com (X.W.); zhangmeng199628@163.com (M.Z.); xiebaohui118@163.com (B.X.); jiangxn@bjfu.edu.cn (X.J.); 2 National Engineering Laboratory for Tree Breeding, The Tree and Ornamental Plant Breeding and Biotechnology Laboratory of Chinese Forestry Administration, Beijing 100083, China

**Keywords:** dehydrin, osmotic stress, DNA binding, *Larix kaempferi*

## Abstract

Dehydrins (DHN) belong to the late embryogenesis abundant II family and have been found to enhance plant tolerance to abiotic stress. In the present study, we reported four DHNs in *Larix kaempferi* (LkDHN) which were identified from the published transcriptome. Alignment analysis showed that these four LkDHNs shared close relationships and belonged to SK_3_-type DHNs. The electrophoretic mobility shift assay indicated that these four LkDHNs all possess sequence-independent binding capacity for double-strands DNAs. The subcellular localizations of the four LkDHNs were in both the nucleus and cytoplasm, indicating that these LkDHNs enter the nucleus to exert the ability to bind DNA. The preparation of tobacco protoplasts with different concentrations of mannitol showed that LkDHNs enhanced the tolerance of plant cells under osmotic stress. The overexpression of LkDHNs in yeasts enhanced their tolerance to osmotic stress and helped the yeasts to survive severe stress. In addition, LkDHNs in the nucleus of salt treated tobacco increased. All of these results indicated that the four LkDHNs help plants survive from heavy stress by participating in DNA protection. These four LKDHNs played similar roles in the response to osmotic stress and assisted in the adaptation of *L*. *kaempferi* to the arid and cold winter of northern China.

## 1. Introduction

Plants are subject to various natural adverse environmental stress, and abiotic stress, such as drought, salinity, high temperature, and cold, which have a significant impact on the growth of plants, resulting in poor plant growth and even death [1,2]. Plants also have evolved a variety of mechanisms to cope with the abiotic stress, including late embryogenesis abundant (LEA) proteins. Dehydrins (DHN) belong to the LEA II family, which accumulates in large amounts in the late stage of seed development when self-induced dehydration occurs [3,4]. DHNs are so named based on their overexpression during seed dehydration stress, which is related to the protective mechanism of plant dehydration [5].

According to the presence of three conserved motifs (Y-, S-, and K-segments), dehydrin proteins are divided into different architectures: Y_n_K_n_, Y_n_SK_n_, K_n_S, SK_n_, and K_n_ [6]. The K segment, a lysine-rich sequence motif (EKKGIMDKIKEKLPG), is prevalent in all dehydrins except in maritime pine and is thought to be a signature fragment [7,8]. Under adversity, the K segment of the dehydrin protein can form a highly conserved alpha helix with both hydrophobic and hydrophilic properties [9]. The Y segment is a conserved sequence [V/T]D[E/Q]YGNP that is similar to the sequence of the nucleotide binding site of plant or bacterial chaperones [10]. Some dehydrin proteins contain S segments, which are composed of serine residues (SSSSSSSD) and can be phosphorylated [11,12].

Many studies have shown that DHNs play an important role in the response to abiotic stress. Overexpressed *MdoDHN11* (*Malus × domestica Borkh.*) can enhance the tolerance of *Arabidopsis thaliana* to drought [13]. The overexpression of the *ShDHN in Solanum habrochaites* enhanced the tolerance of transgenic tomatoes to multiple abiotic stress [14]. Although the mechanism of its function has not been clearly resolved, some models have been proposed to elucidate the role of dehydrins in abiotic stress. Dehydrins can bind metal ions, DNA, protein, or membranes to protect cells from various environmental stress. AtHIRD11 (*A. thaliana*) and CuCOR15 (*Citrus unshiu*) dehydrins can bind iron, nickel, copper, and zinc through magnesium and calcium ions to reduce the production of reactive oxygen species (ROS) [15,16]. VrDHN1 in *Vigna radiate* showed low affinity for nonspecific interactions with DNA [17]. OsDHN-rab16d (*Oryza sativa*) combines with OsFKBP (a prolyl *cis-trans* isomerase) to form a complex that is involved in the signal transduction of the abscisic acid (ABA) response to drought stress in rice [18]. The Lti30 dehydrin in *A.thaliana* interacts with the membrane electrostatically where anionic lipid headgroups are exposed [19,20].

*Larix kaempferi* is the main afforestation tree species in northern China, which is characterized by arid and cold in winters [21]. Studying the molecular mechanisms of *L. kaempferi* resistance to stress is important for improving its resistance to stress. DHNs have been reported to be involved in the resistance to osmotic stress and are related to the protective mechanism of plant dehydration [5]. However, there is currently little research on the DHNs of *L. kaempferi*. In this study, we identified and cloned the *DHN* genes from *L. kaempferi* (LkDHN) based on the published transcriptome of *L. laricina* FK-6-B (SRX4092599). Through subcellular localization and electrophoretic mobility shift assay (EMSA) experiments, it was found that LkDHNs have the ability to bind DNA, and the roles of different LkDHNs in osmotic resistance were determined using osmotic stress experiments on tobacco protoplasts and yeast strains overexpressing of LkDHNs.

## 2. Results

### 2.1. Transcriptome Based Screening and Cloning of DHN Genes in Larix Kaempferi

Transcriptome-based screening of DHN homologs was firstly performed using *Pinus massonia* PmDHN (KF910087) as a query against the *L. laricina* FK-6-B transcriptome. Four candidate DHNs (TRINITY_DN53859_c1_g6_i6, TRINITY_DN53859_c1_g6_i3, TRINITY_DN53859_c1_g6_i4, and TRINITY_DN53859_c1_g6_i14) were identified as the DHN ortholog transcripts in *L. laricina* with distinct high scores, identity >50%, and a low E-value (E-20). Then, the LkDHN1 (MK211162), LkDHN2 (MW349029), LkDHN3 (MW349030), and LkDHN4 (MW349031) were cloned and the sequences were deposited in GenBank. The length of the LkDHNs CDS ranged from 585 to 609 bp and encoded 194 to 202 amino acids (Table S1: Analysis of physical and chemical properties of LkDHNs).

### 2.2. Alignment Analysis of DHNs in Plants

**Figure 1:**
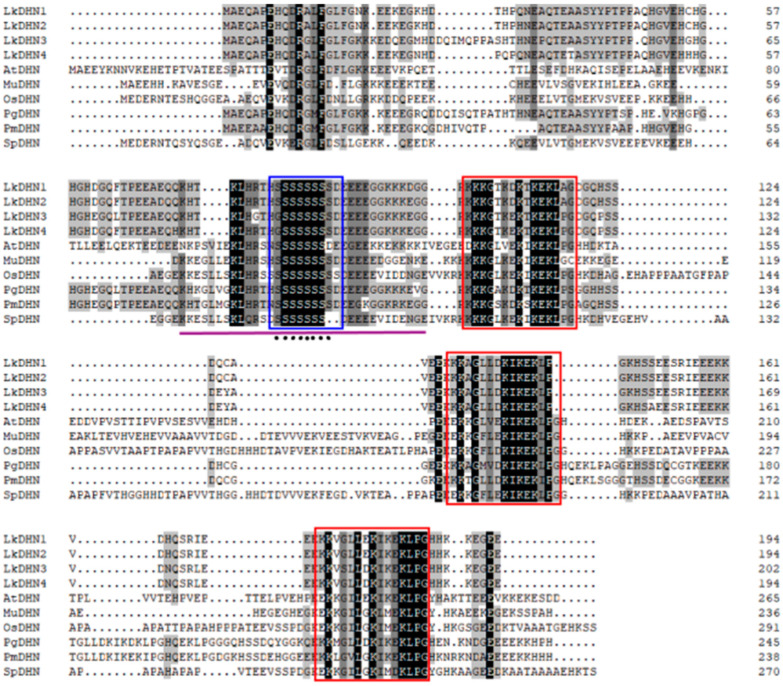
Alignment of LkDHNs (Dehydrin in *Larix kaempferi*) and DHNs from other plants. Six verified DHNs including AtDHN (*Arabidopsis thaliana*), MnDHN (*Musa nana*), OsDHN (*Oryza sativa*), PgDHN (*Picea glauca*), PmDHN (*Pinus massonia*), SpDHN (*Stipa purpurea*) and LkDHNs homologous sequence were aligned by DNAman 8.0. The S segment was indicated by blue box; K segments were indicated by red boxes; nuclear localization signals (NLS) were indicated by the purple line; phosphorylation sites were indicated by black dot.

Alignment of LkDHNs and reference DHNs from different plants was performed. Compared with reported DHNs in *A. thaliana*, *O.sativa*, and *P.massonia*, the highly conserved motifs were detected in all aligned DHNs. A serine-rich S segment was found in all four LkDHNs. In addition, three K segments were also detected in LkDHNs (Figure 1). The above results confirmed that the four cloned LkDHNs belong to DHNs in gymnosperms and are SK_3_-type DHNs. As showed in Figure 1, the analysis of nuclear localization signals (NLS) showed that LkDHNs all have NLS sequences (KHTKLH(R/G)THSSSSSSSSDEEEEGGKKKDGG). The prediction of phosphorylation sites showed that eight serines in S segment could be phosphorylated.

### 2.3. Subcellular Localization of LkDHNs

**Figure 2:**
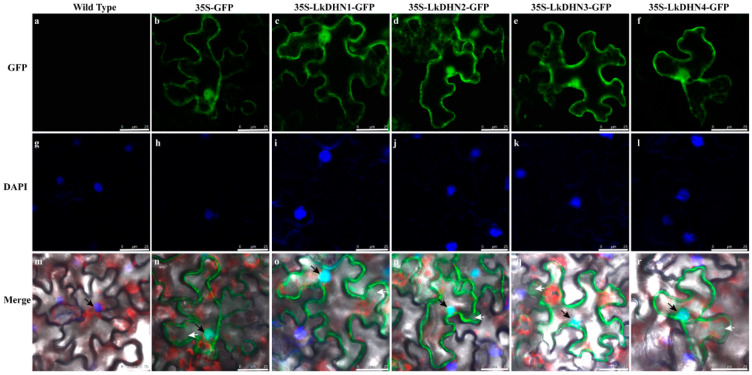
Subcellular localization of LkDHNs in lower epidermis cells of the transformed tobacco leaves. (**a**–**f**) are wild type, 35S-GFP, 35S-LkDHN1-GFP, 35S-LkDHN2-GFP, 35S-LkDHN3-GFP, and 35S-LkDHN4-GFP at the wavelength of GFP, respectively; (**g**–**l**) are wild type, 35S-GFP, 35S-LkDHN1-GFP, 35S-LkDHN2-GFP, 35S-LkDHN3-GFP, and 35S-LkDHN4-GFP at the wavelength of DAPI, respectively; (**m**–**r**) are the merge of wild type, 35S-GFP, 35S-LkDHN1-GFP, 35S-LkDHN2-GFP, 35S-LkDHN3-GFP, and 35S-LkDHN4-GFP, respectively. The nucleuses were indicated by black arrow; the cytoplasms were indicated by white arrow. The scale bar is 25 μm.

To explore the subcellular localization of LkDHNs, the pBI121-LkDHNs-GFP recombinant vectors were constructed and transferred to *Agrobacterium tumefaciens* GV3101, then transformed to tobacco leaves. Compared with wild-type (Figure 2a,g,m) and 35S-GFP (Figure 2b,h,n), LkDHNs could detect GFP fluorescence signals in the nucleus and cytoplasm of lower epidermis cells of tobacco leaves at the wavelength of GFP. In addition, the GFP fluorescence signals on the nucleus were found to coincide with the 4, 6-diamidino-2-phenylindole (DAPI) signals. Based on the green fluorescent protein (GFP) signals, four LkDHNs were found to mainly locate in the nucleus and also exist in the cytoplasm (Figure 2).

### 2.4. Stress Treatment Enhanced the Signal of LkDHNs in Nucleus

**Figure 3:**
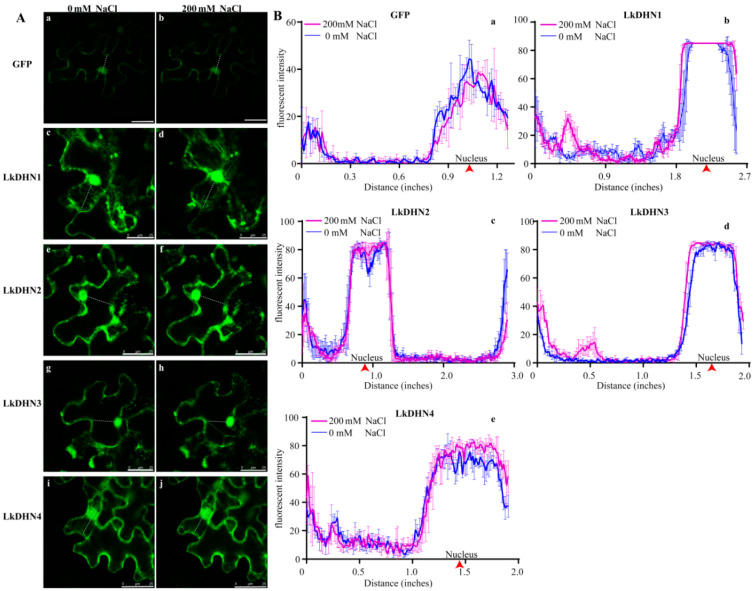
Stress treatment enhanced the signal of LkDHNs in nucleus. (**A**) The effect of salt stress on the nuclear localization of LkDHNs was observed by a Leica SP8 laser confocal microscope. **a** and **b** are GFP with 0 and 200 mM NaCl, respectively; **c** and **d** are LkDHN1 with 0 and 200 mM NaCl, respectively; **e** and **f** are LkDHN2 with 0 and 200 mM NaCl, respectively; **g** and **h** are LkDHN3 with 0 and 200 mM NaCl, respectively; **i** and **j** are LkDHN4 with 0 and 200 mM NaCl, respectively. (**B**) Analysis of GFP fluorescence signal in nucleus under salt stress. **a**: GFP; **b**: LkDHN1; **c**: LkDHN2; **d**: LkDHN3; **e**: LkDHN4. The purple line represents the fluorescence after 30 min treatment with 200 mM NaCl, and the blue line represents the fluorescence under 0 mM NaCl. The nucleuses were indicated by red arrow.

The transient transformed tobacco leaves were treated with 200 mM NaCl. As shown in Figure 3, the fluorescence intensity in nucleus of GFP treated with NaCl was lower than that of untreated, while the fluorescence intensity in the nucleus of LkDHNs treated with NaCl was slightly stronger than that of untreated, indicating that LkDHNs could play a role in the plant cell nucleus under stress.

### 2.5. LkDHNs Bind Double-Stranded DNAs

**Figure 4:**
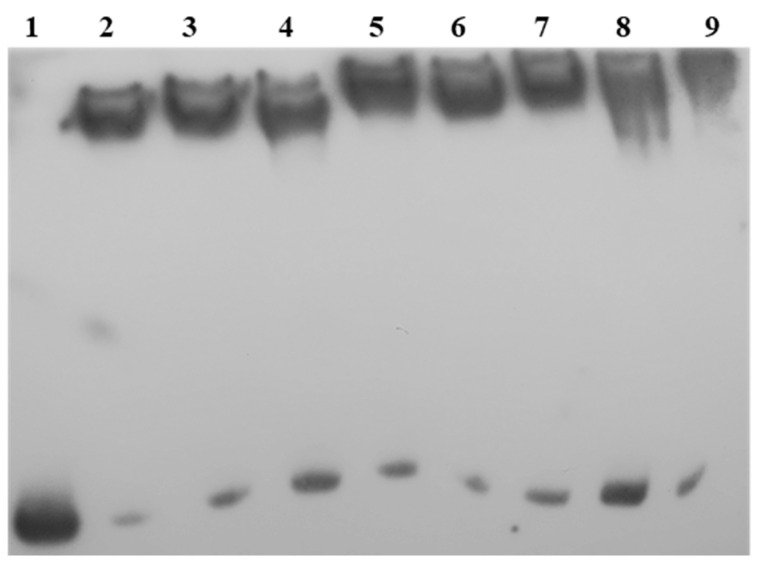
The binding ability of LkDHNs to different DNA probes was analyzed by electrophoretic mobility shift assay (EMSA). Lane 1: free-probe; Lane 2: CSE-probe with LkDHN1; Lane 3: CSE-probe with LkDHN2; Lane 4: CSE-probe with LkDHN3; Lane 5: CSE-probe with LkDHN4; Lane 6: pET28a-probe with LkDHN1; Lane 7: pET28a-probe with LkDHN2; Lane 8: pET28a-probe with LkDHN3; Lane 9: pET28a-probe with LkDHN4.

To validate the capacity of LkDHNs to bind double-stranded DNAs (dsDNAs), EMSA was performed using recombinant LkDHNs and different dsDNAs (CSE-probe and pET28a-probe). The results showed that all four LkDHNs could bind the dsDNAs without sequence specificity (Figure 4). This sequence-independent binding capacity of LkDHNs guarantees that LkDHNs can execute functions in the nucleus, such as protecting DNA from damage.

### 2.6. Overexpression of LkDHNs Increased the Tolerance of Tobacco Protoplasts and Yeasts to Osmotic Stress

**Figure 5:**
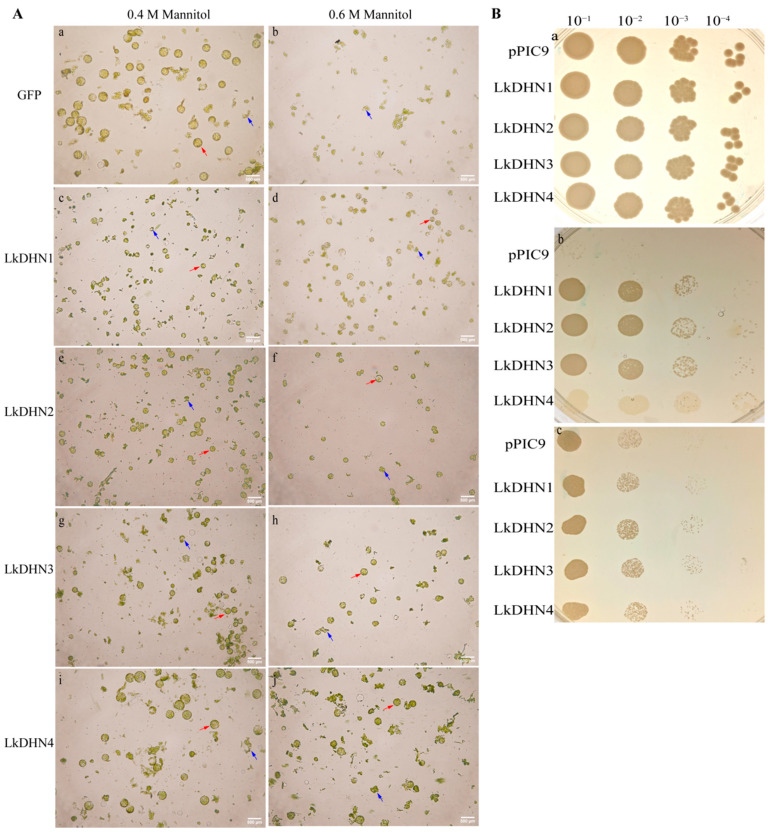
LkDHNs increased the tolerance of tobacco protoplasts and yeasts to osmotic stress. (**A**) The tolerance of tobacco protoplasts with LkDHNs to osmotic stress was enhanced. **a** and **b** are the enzymatic hydrolysis of GFP at 0.4 and 0.6 M mannitol, respectively; **c** and **d** are the enzymatic hydrolysis of LkDHN1 at 0.4 and 0.6 M mannitol, respectively; **e** and **f** are the enzymatic hydrolysis of LkDHN2 at 0.4 and 0.6 M mannitol, respectively; **g** and **h** are the enzymatic hydrolysis of LkDHN3 at 0.4 and 0.6 M mannitol, respectively; **i** and **j** are the enzymatic hydrolysis of LkDHN4 at 0.4 and 0.6 M mannitol, respectively. The intact protoplasts were indicated by red arrow; the broken protoplasts were indicated by blue arrow. The scale bar is 500 μm. (**B**) Enhanced tolerance of yeast overexpressing LkDHNs to osmotic stress. **a**: spot assay of pPIC9 and LkDHNs on YPD medium; **b**: spot assay of pPIC9 and LkDHNs on YPD medium with 2 M sorbitol; **c**: spot assay of pPIC9 and LkDHNs on YPD medium with 1.5 M NaCl.

The tobacco leaves transiently transfected with GFP and LkDHNs, and then enzymatically hydrolyzed with different concentrations of mannitol to obtain protoplasts. Many protoplasts could be obtained from GFP and LkDHNs at 0.4 M mannitol. However, when the mannitol concentration increased to 0.6 M, the protoplasts of GFP were broken due to high osmotic stress, while the protoplasts of LkDHNs were still intact (Figure 5A). These results indicated that LkDHNs can improve the tolerance of plant cells to osmotic stress.

Yeasts overexpressing LkDHNs showed no obvious difference in growth compared with the control (yeast with the empty vector pPIC9) in yeast extract-peptone-dextrose (YPD) with or low concentration sorbitol. As showed in Figure 5B, when the YPD medium contained 2 M sorbitol, the survival rate of LkDHNs was significantly higher than that of the control. But the tolerance of LkDHN4 to oxidative stress was not as high as LkDHN1-3. When the medium contained 1.5 M NaCl, LkDHNs grew better than the control, especially in the lane of 10^−3^ dilution. This result indicated that the overexpression of LkDHNs in yeast enhanced the tolerance to osmotic stress and could enable the survival of yeast under heavy osmotic stress.

## 3. Discussion

The functions of dehydrins in plant tolerance have been widely studied under a variety of osmotic stress. However, there are few reports on the DHNs in *L. kaempferi* and related information. In this study, we identified and cloned the *DHN* genes from *L. kaempferi* based on the published transcriptome. In the recently reports, the localization of dehydrins have shown that DHNs are located in many parts of cells, such as the cytoplasm, nucleus, chloroplast, vacuole, endoplasmic reticulum, mitochondria, cytoplasm, and cell membrane [22]. Studies have also shown that dehydrin protein containing S segment composed of serine can be phosphorylated and transferred to the nucleus to make function [5,23]. The prediction of phosphorylation sites of LkDHNs suggested that the S segment could be phosphorylated. Additionally, subcellular localization of LkDHNs indicated that they were located in the nucleus and cytoplasm. So, it is possible that LkDHNs enter the nucleus to exert the ability after being phosphorylated.

Salt stress of transient transformed tobacco leaves showed that the fluorescence intensity of LkDHNs in the nucleus increased. Using EMSA, it was found that LkDHNs could bind to different probes, which were nonspecific. Dehydrins were reported to have the function of binding DNA to protect DNA from damage caused by environmental stress. Thus far, some studies have reported that DHN proteins can bind to DNA, the *Vitis riparia* dehydrin locates in the nucleus and binds to DNA to protect it from hydrogen peroxide [24]. Given that the four LkDHNs could bind the dsDNAs and present in both the nucleus and cytoplasm, it is possible that these LkDHNs were located in the nucleus to protect the DNA from oxidative stress. The KS type CuCOR15 needs zinc ions to bind to DHN, but Y_2_K type VrDHN1 does not need metal ions to bind to DNA [16,17]. Lin et al. suggested that the absence of Y fragment resulted in that CuCOR15 needed Zn^2+^ to bind to DNA [17]. However, LkDHNs are SK_3_ type dehydrin, and do not add metal ions when binding to DNA. Therefore, the mechanism of LkDHNs may be slightly different, but it needs further study.

To explore whether LkDHNs exhibit tolerance to osmotic stress, the LkDHNs were transferred into yeast cells and tobacco protoplasts for stress treatment. In the presence of high concentration of mannitol, the protoplasts of GFP were broken, while the protoplasts of LkDHNs were intact, indicating that LkDHNs can improve the stress resistance of plant cells. Compared with the control, yeast strains overexpressing LkDHNs grew better in YPD containing 2 M sorbitol and 1.5 M NaCl. These results indicate that LkDHNs can enhance the tolerance of yeast cells to osmotic stress and may play a protective role in plants under stress. Similarly, overexpressing *CaDHN5* (*Capsicum annuum*) in *A.thaliana* enhanced its tolerance to salt and osmotic stress, and the expression levels of genes related to salt and osmotic stress were also increased [24]. The *IbLEA14* gene in *Ipomoea batatas* enhances the salt tolerance and osmotic stress tolerance of callus [25]. All these results indicate the four LkDHNs are important for plants to survive severe stress by participating in DNA protection.

## 4. Materials and Methods

### 4.1. Plant Materials

Two-year-old *L. kaempferi* strains were harvested as plant materials from Liaoning province, China. Collected samples were immediately frozen in liquid nitrogen and stored at −80 °C for future use.

### 4.2. Transcriptome Based Identification of DHN Genes in L. kaempferi

According to published papers, the DHN of *P. massoniana* was retrieved in the NCBI database. PmaDHN (KF910087) was used as the query sequence, and tBlastn was performed on the published *Larix laricina* transcriptome (SRX4092599) to search for the homologous sequence of *L. kaempferi* DHN. Based on the retrieved sequence information, primers were designed and the *DHN* gene of *L. kaempferi* was cloned (Table S2: Information on primers.).

### 4.3. DHN Sequence Alignments

Using DNAman 8.0, six verified DHNs including AtDHN, MnDHN (*Musa nana*), OsDHN, PgDHN (*Picea glauca*), PmDHN, SpDHN (*Stipa purpurea*), and LkDHNs homologous sequence were aligned [26,27]. Motifs were detected by submitting LkDHNs to Pfam (http://pfam.janelia.org (accessed on 1 December 2020)). The NLS of LkDHNs were obtained by cNLS Mapper (http://nls-mapper.iab.keio.ac.jp/cgi-bin/NLS_Mapper_form.cgi (accessed on 1 December 2020)). The prediction of phosphorylation sites was determined by NetPhos 3.1 Server (http://www.cbs.dtu.dk/services/NetPhos/ (accessed on 1 December 2020)). The DHN sequences used in bioinformatics analysis are listed in Table S3: DHN information for alignment and phylogentic analysis.

### 4.4. Subcellular Location of LkDHNs

The pBI121 vector with the LkDHNs and GFP fusion expression was constructed, and the enzyme restriction sites selected for this vector were *Xba*I and *Bam*HI. The *Agrobacterium tumefaciens* strain GV3101 transformed with the recombinant plasmid was cultured in YEB liquid medium containing 50 mg/L rifampicin and 50 mg/L kanamycin at 28 °C overnight. Fifty microliters of bacterial solution was removed and added to the YEB medium until the OD_600_ was 0.4–0.6. Two milliliters of the bacterial solution was pipetted and centrifuged at 8000 rpm for 90 s to collect the bacterial cells. The introduction solution (53 mM MES pH 5.6, 29 mM glucose, 2.1 mM NaH_2_PO_4_) and 20 × AB salt solution (374 mM NH_4_Cl, 24.3 mM MgSO_4_·7H_2_O, 40.2 mM KCl, 180 µM FeSO_4_·7H_2_O, 1.36 mM CaCl_2_·2H_2_O) were added at a ratio of 19:1 to resuspend the bacterial pellet. *Nicotiana benthamiana* leaves were infiltrated with resuspension by syringe and were cultured under dark conditions for 16 h, following which they were moved to light for 48 h. The tobacco leaves were cut to 1 cm × 1 cm and dyed in DAPI solution (Beyotime, Shang Hai, China) for 1 h. The nuclear localization of LkDHNs was observed in leaves treated with 200 mM NaCl for 30 min. The GFP fluorescence was detected by a Leica SP8 laser confocal microscope (Leica Microsystems, Wetzlar, Germany) and observed using an excitation spectrum of 488 nm and emission spectrum of 507 nm. The DAPI was observed using an excitation spectrum of 364 nm and emission spectrum of 454 nm.

### 4.5. The Binding Ability Analysis of LkDHNs and DNA by Electrophoretic Mobility Shift Assay

The pET28a-LkDHNs vectors were constructed using the Onestep Seamless Kloning kit (Aidlab, Beijing, China) and transformed into *E.coli* strain BL21 (DE3). The recombinant strain was cultured overnight at 37 °C in lysogeny broth (LB) medium containing 500 mg/L kanamycin, and cultured in 1 L LB medium at a ratio of 1:20 until the OD_600_ reached 0.6, following which 0.4 mM isopropyl β- d-thiogalactoside (IPTG) was used to induce protein expression at 28 °C for 4 h. For protein purification, the recombinant protein was washed with a wash buffer (50 mM Tris-HCl pH 8.0, 300 mM NaCl, and 20 mM imidazole) and then eluted with an elution buffer (50 mM Tris-HCl pH 8.0, 300 mM NaCl, and 150 mM imidazole) [28]. The partial sequence of the caffeoyl shikimate esterase (CSE) (MK211161) and the partial end of pET28a vector (about 200 bp) were selected as probes and labeled by a Beyotime EMSA probe biotin labeling kit (Beyotime, Shang Hai, China). To verify the binding ability of the DHNs with different DNAs, different probes were mixed with 1 μg of LkDHN1, LkDHN2, LkDHN3, and LkDHN4 proteins, respectively, and then subjected to polyacrylamide gel electrophoresis at 100 V for 1 h and transferred to a nylon membrane by electrophoretic transfer. After that, UV cross-linking was carried out for 10 min to fix the DNA on the membrane, and the probe was detected by a Beyotime chemiluminescent biotin-labeled nucleic acid detection kit (Beyotime, Shang Hai, China).

### 4.6. Preparation of Tobacco Protoplasts by Enzymatic Hydrolysis

After transient expression, the leaves were selected and the lower epidermises were removed. About 30 mg leaves were cut into small pieces and then immersed in 1 mL enzymolysis solution {1.5% cellulase, 0.3% macerozyme R-10, 0.4 M, or 0.6 M mannitol, 20 mM KCl, 20 mM 2-(N-morpholino) ethanesulfonicacid (MES), 10 mM CaCl_2_, 0.1% bovine albumin (BSA)} for 40 min at 40 rpm at room temperature. After enzymolysis, the protoplasts were allowed to settle at room temperature for 10 min, and then the enzymolysis solution was carefully discarded. Twenty-five microliters of W5 solution (125 mM CaCl_2_, 154 mM NaCl, 5 mM KCl, 5 mM glucose, 2 mM MES, pH 5.6) was added for resuspension, and 15 μL protoplasts were taken for observation under the microscope (Olympus, Tokyo, Japan).

### 4.7. Stress Resistance Assay of LkDHNs in Yeast Transformants

The pPIC9-LkDHNs vectors were constructed and transformed into *Pichia pastoris* GS115, and high-expression strains were screened. The pPIC9 and pPIC9-LkDHNs yeast strains were cultured in buffered complex glycerol medium (BMGY) at 30 °C 250 rpm for 36 h until OD_600_ = 2–3, following which they were induced and cultured with buffered methanol complex medium (BMMY) containing 0.5% methanol for 72 h, with methanol added to the medium every 24 h. The OD_600_ value in the induced strains was detected, and the strains were diluted to OD_600_ = 1, and then diluted by 10, 100, 1000, and 10,000 times [29]. Four microliters of bacterial solution was taken and spotted on YPD medium containing 0 M and 2 M sorbitol or 0 M. and 1.5 M NaCl, and incubated at 30 °C for 72 h.

## 5. Conclusions

Dehydrins belonging to the LEA II family have been reported to participate in the response to osmotic stress, such as drought and cold stress. However, DHN in *L. kaempferi* have been little explored. Based on the published transcriptome, four SK_3_-type DHNs in *L. kaempferi* were characterized, and functional analysis elucidated their roles in tolerating with osmotic stress. These four LkDHNs shared high sequence identity and showed functional similarity in yeasts and tobacco protoplasts. The overexpression of any LkDHNs in yeast could help the yeasts survive from severe osmotic stress. The protoplasts of LkDHNs can tolerate high osmotic stress. The important roles of LkDHNs in the response to osmotic stress are likely based on the capacity of LkDHNs to bind dsDNAs. This sequence-independent binding capacity of LkDHNs helped the plants or yeasts survive the DNA damages caused by severe osmotic stress. Further work should be performed in the future to explore the mechanisms of LkDHNs in DNA protection under osmotic stress.

## Data Availability

The data presented in this study are available in article and Supplementary Materials.

## Supplementary Materials

The following are available online at https://www.mdpi.com/1422-0067/22/4/1715/s1.

## Abbreviations

BMGY	Buffered complex glycerol medium
BMMY	Buffered methanol complex medium
BSA	Bovine albumin
CSE	Caffeoyl shikimate esterase
DAPI	4,6-diamidino-2-phenylindole
dsDNAs	Double-stranded DNAs
EMSA	Electrophoretic mobility shift assay
GFP	Green fluorescent protein
LB	Lysogeny broth medium
LEA	Late embryogenesis abundant protein
MES	2-(N-morpholino) ethanesulfonicacid
NLS	nuclear localization signals
ROS	Reactive oxygen species
YPD	Yeast extract-peptone-dextrose
