# Supplementary material for: Functional Characteristics Analysis of Dehydrins in Larix kaempferi under Osmotic Stress
Source: Int J Mol Sci. 2021 Feb 9;22(4):1715. doi: 10.3390/ijms22041715 (PMC7915896; doi:10.3390/ijms22041715)
Supplement: Supplementary file 1 [file ijms-22-01715-s001.pdf]

Table S1. Analysis of physical and chemical properties of LkDHNs.

gene name	Gene length(bp)	Number of amino acids	Molecular weight (kDa)	Isoelectric point
LkDHN1	585	194	21.7	6.26
LkDHN2	585	194	21.7	6.29
LkDHN3	609	202	22.6	5.94
LkDHN4	585	194	21.8	6.12

Table S2. Information on primers.

Primer Name	Sequence(5'-3')
LkDHN1 F：	ATGGCTGAACAAGCACCA
LkDHN1 R：	CTCTTCCCCTTCCTTCTT
LkDHN2 F：	ATGGCTGAACAAGCACCA
LkDHN2 R：	CTCTTCCCCTTCCTTCTT
LkDHN3 F：	ATGGCCGAACAAGCGCCG
LkDHN3 R：	CTCTTCCCCTTCCTTCTT
LkDHN4 F：	ATGGCTGAACAAGCACCA
LkDHN4 R：	CTCTTCCCCTTCCTTCTT
pET28a-LkDHN1 F:	CAGCAAATGGGTCGCGGATCCATGGCTGAACAAGCACCA
pET28a-LkDHN1 R:	GCAAGCTTGTCGACCGAGCTCTTACTCTTCCCCTTCCTTCTT
pET28a-LkDHN1 F:	CAGCAAATGGGTCGCGGATCCATGGCTGAACAAGCACCA
pET28a-LkDHN1 R:	GCAAGCTTGTCGACCGAGCTCTTACTCTTCCCCTTCCTTCTT
pET28a-LkDHN1 F:	CAGCAAATGGGTCGCGGATCCATGGCTGAACAAGCACCA
pET28a-LkDHN1 R:	CAGCAAATGGGTCGCGGATCCATGGCCGAACAAGCGCCG
pET28a-LkDHN1 F:	CAGCAAATGGGTCGCGGATCCATGGCTGAACAAGCACCA
pET28a-LkDHN1 R:	GCAAGCTTGTCGACCGAGCTCTTACTCTTCCCCTTCCTTCTT
pBI121-LkDHN1-GFP F:	TGGAGAGAACACGGGGGACTCTAGAATGGCTGAACAAGCACCA
pBI121-LkDHN1-GFP R	TCACCATACCTCCTCCTCCGGATCCCTCTTCCCCTTCCTTCTT
pBI121-LkDHN2-GFP F:	TGGAGAGAACACGGGGGACTCTAGAATGGCTGAACAAGCACCA′
pBI121-LkDHN2-GFP R:	TCACCATACCTCCTCCTCCGGATCCCTCTTCCCCTTCCTTCTT
pBI121-LkDHN3-GFP F:	TGGAGAGAACACGGGGGACTCTAGAATGGCCGAACAAGCGCCG′

pBI121-LkDHN3-GFP R:	TCACCATACCTCCTCCTCCGGATCCCTCTTCCCCTTCCTTCTT
pBI121-LkDHN4-GFP F:	TGGAGAGAACACGGGGGACTCTAGAATGGCTGAACAAGCACCA’
pBI121-LkDHN4-GFP R:	TCACCATACCTCCTCCTCCGGATCCCTCTTCCCCTTCCTTCTT
CSE-probe F:	CCCACGGCATCCTCTTCACG
CSE-probe R:	CGGCGATCTTCTCCATGTCTCC
pET28a-probe F:	ATGTTATATCCCGCCGTTAACC
pET28a-probe R:	AATGAATCGGCCAACGC

Table S3. DHN information for alignment analysis.

Gene Id	Species	Symbol	Platform
KP234263.1	*Pinus massoniana*	PmaCSE	NCBI
AAD28175	*Picea glauca*	PgDHN	NCBI
KJ000690.1	*Stipa purpurea*	SpDHN	NCBI
JF320824	*Musa nana*	MuDHN	NCBI
ABS44866	*Oryza sativa*	OsDHN	NCBI
CAA62449	*Arabidopsis thaliana*	AtDHN	NCBI
